# Golimumab for the treatment of refractory juvenile idiopathic arthritis-associated uveitis

**DOI:** 10.1007/s12348-012-0081-y

**Published:** 2012-05-14

**Authors:** Mridula William, Sepideh Faez, George N. Papaliodis, Ann-Marie Lobo

**Affiliations:** Massachusetts Eye and Ear Infirmary, 243 Charles Street, Boston, MA 02114 USA

## Introduction

Juvenile idiopathic arthritis (JIA)-associated uveitis is a potentially blinding disease and accounts for up to 75 % of all pediatric anterior uveitis cases in many tertiary care referral centers [1]. Inability to control ocular inflammation in pediatric uveitis can lead to complications such as cataracts, macular edema, band keratopathy, and glaucoma leading to vision loss [1].

Off-label use of biologic response modifiers such as tumor necrosis factor alpha (TNFα) inhibitors, including infliximab, adalimumab, and etanercept, have expanded the treatment armamentarium for refractory JIA-associated uveitis. A cross-sectional survey by Foeldvari et al. demonstrated that TNF inhibitors were effective in two thirds of patients with JIA-associated uveitis who did not respond to conventional immunosuppressive agents [2].

Golimumab (GLM) is a novel fully humanized anti-TNFα monoclonal antibody that has been approved for the treatment of rheumatoid arthritis, psoriatic arthritis, and ankylosing spondylitis [3]. We present three cases of refractory JIA-associated uveitis treated with GLM. Institutional review board approval for this case series was waived by the Massachusetts Eye and Ear Infirmary Human Studies Committee.

## Case 1

A 15-year-old girl was diagnosed with JIA-associated uveitis at the age of 6. Treatment included infliximab and methotrexate for 7 years with control of ocular inflammation. Infliximab was discontinued with subsequent recurrence of inflammation. She developed a severe allergic reaction upon resuming infliximab. Other therapies attempted for ongoing inflammation included mycophenolate mofetil (ineffective), adalimumab (side effect of palpitations), azathioprine (ineffective), and daclizumab (medication discontinued by manufacturer). On presentation to our institution, best corrected visual acuity (BCVA) was 20/70 in the right eye (OD) and 20/25 in the left (OS). Slit lamp exam demonstrated 1+ anterior chamber cell OD (based on Standard Uveitis Nomenclature criteria) and 2+ anterior chamber cell OS with posterior synechiae OS [4]. Intraocular pressures were normal. There were early posterior subcapsular cataracts bilaterally and fundus exam was unremarkable. Given continued active inflammation on oral corticosteroids and methotrexate, GLM therapy was initiated at 50 mg subcutaneously once per month with quiescence of ocular and joint disease within 2 months of starting therapy. The patient required GLM every 3 weeks in order to control ocular inflammation that developed prior to the next scheduled dose. Methotrexate dose was also maximized to 25 mg per week subcutaneously, and topical steroids four times daily were continued. Oral corticosteroids were tapered and discontinued. The patient developed visually significant posterior subcapsular cataracts due to prolonged inflammation and corticosteroid therapy with a decline in BCVA to less than 20/200 in both eyes (OU). Uveitis was quiescent for 3 months prior to cataract surgery. Clear corneal incision cataract surgery was performed OU with intracameral preservative-free triamcinolone and intravenous methylprednisolone at the time of surgery. Subsequently, she underwent YAG capsulotomy with improvement in BCVA to 20/60 OU and quiescence of inflammation on GLM therapy for a duration of 18 months.

## Case 2

A 27-year-old man was diagnosed with JIA uveitis at age 2 and polyarthritis at age 5. He was treated with steroid drops and methotrexate for chronic uveitis. He developed recurrent inflammation as an adult and failed therapy with etanercept and adalimumab. Infliximab was added at age 24. The patient’s ocular inflammation was controlled for 2 years until he started experiencing unusual flares of arthritis following infliximab infusions thought to be medication induced and requiring discontinuation of infliximab. GLM 50 mg subcutaneously once per month was added to the existing regimen of weekly subcutaneous 25 mg methotrexate. BCVA was 20/20 OU and there was no active uveitis on slit lamp examination. Fundus exam was unremarkable. The patient continued to have symptoms of joint inflammation and developed a uveitis flare with 3+ cells OU after 6 months of GLM therapy. BCVA was 20/50 OD and 20/25 OS. Because of worsening of eye and arthritis symptoms on GLM, infliximab therapy was reinstituted along with mycophenolate mofetil. The patient continued to have flares of joint inflammation on this therapy and required pulse dose methylprednisolone. Tocilizumab infusions were attempted, but the patient had a flare of uveitis following this therapy and infliximab infusions were reinstituted along with periocular corticosteroid injections.

## Case 3

A 10-year-old girl was diagnosed with JIA at the age of 18 months and treated with methotrexate and etanercept which was switched to adalimumab when she developed uveitis. She presented to our institution at age 7 for refractory JIA-associated uveitis OS. The patient also had treatment failure with abatacept and an allergic reaction to infliximab. Because of worsening of ocular and joint symptoms, GLM therapy was added to the existing regimen of weekly subcutaneous methotrexate (25 mg) and once daily topical steroid. BCVA was 20/25 OD and 20/400 OS. IOP was normal OU. There was 3+ cell with posterior synechiae and a posterior subcapsular cataract OS. One month after starting GLM, there was quiescence of inflammation OS with improvement in visual acuity to 20/200. Her joint inflammation also improved. After 4 months of quiescence, she underwent cataract surgery OS with intracameral preservative-free triamcinolone. She developed iris bombe with pupillary block following surgery requiring a surgical iridectomy. Best corrected visual acuity improved to 20/80, intraocular pressure normalized to 16 mmHg off anti-glaucoma medications, and inflammation was quiet within 1 month after surgery.

## Discussion

We present the use of GLM in refractory JIA uveitis. To our knowledge, there is only one report in the literature on the use of GLM in uveitis which included an adult JIA uveitis patient and a retinal vasculitis patient who failed other TNF inhibitor therapies [5].

Various immunomodulatory drugs have been used to treat uveitis in children to minimize ocular complications that may result in vision loss. A number of observational studies have shown the benefits of anti-TNF agents in the treatment of childhood uveitis [2]. GLM has been well tolerated in clinical trials with a safety profile comparable to other currently available TNFα inhibitors [3, 6]. One advantage of GLM over other TNF inhibitors is its subcutaneous once a month dosing schedule. GLM avoids the more frequent dosing required of some TNF inhibitors such as adalimumab and eliminates the expense and time commitment of outpatient infusions required for other TNF inhibitors such as infliximab. GLM is a fully humanized monoclonal antibody with lower likelihood of the development of neutralizing antibodies and allergic reaction than infliximab. GLM has also been shown efficacious in patients who have previously failed an anti-TNFα agent [7].

Patient 1 was refractory to multiple medications but achieved long-term quiescence with GLM; the change in the interval dosing schedule for GLM likely reflects the aggressive nature of her disease. Patient 2 may have had an initial response to GLM but then developed recurrence of both ocular and joint symptoms and has failed numerous other therapies, which indicates that GLM may not be superior to other TNF inhibitors for patients with severe disease. Patient 3 failed three TNF inhibitors before GLM therapy was initiated with significant improvement in ocular inflammation, although her follow-up period has only been for 6 months. No patient developed adverse effects from GLM in spite of prior adverse reactions to other TNF inhibitors. Two out of three patients had control of ocular and joint inflammation on GLM therapy and were able to undergo cataract surgery with continued control of inflammation and improvement in visual acuity.

## Conclusion

GLM appears to be safe and provides an alternative treatment in refractory JIA uveitis, including patients who have failed other TNF inhibitors. The authors acknowledge that this is a small retrospective case series, and further studies are required to understand the long-term efficacy and safety of GLM in the treatment of uveitis.
